# Altered organization of collagen fibers in the uninvolved human colon mucosa 10 cm and 20 cm away from the malignant tumor

**DOI:** 10.1038/s41598-020-63368-y

**Published:** 2020-04-14

**Authors:** Sanja Z. Despotović, Đorđe N. Milićević, Aleksandar J. Krmpot, Aleksandra M. Pavlović, Vladimir D. Živanović, Zoran Krivokapić, Vladimir B. Pavlović, Steva Lević, Gorana Nikolić, Mihailo D. Rabasović

**Affiliations:** 1 University of Belgrade, Faculty of Medicine, Institute of Histology and embryology, Belgrade, Serbia; 2 Saarland University, Department of Internal Medicine V- Pulmonology, Allergology, Intensive Care Medicine, Homburg/Saar, Germany; 3 University of Belgrade, Institute of Physics Belgrade, Belgrade, Serbia; 4 University Hospital Center “Dr Dragiša-Mišović-Dedinje”, Belgrade, Serbia; 5 Clinic for Abdominal Surgery- First surgical clinic, Clinical Center of Serbia, Belgrade, Serbia; 6 University of Belgrade, Faculty of Agriculture, Belgrade, Serbia; 7 University of Belgrade, Faculty of Medicine, Institute of Pathology, Belgrade, Serbia

**Keywords:** Cancer, Gastroenterology, Medical research, Molecular medicine, Pathogenesis

## Abstract

Remodelling of collagen fibers has been described during every phase of cancer genesis and progression. Changes in morphology and organization of collagen fibers contribute to the formation of microenvironment that favors cancer progression and development of metastasis. However, there are only few data about remodelling of collagen fibers in healthy looking mucosa distant from the cancer. Using SHG imaging, electron microscopy and specialized softwares (CT-FIRE, CurveAlign and FiberFit), we objectively visualized and quantified changes in morphology and organization of collagen fibers and investigated possible causes of collagen remodelling (change in syntheses, degradation and collagen cross-linking) in the colon mucosa 10 cm and 20 cm away from the cancer in comparison with healthy mucosa. We showed that in the lamina propria this far from the colon cancer, there were changes in collagen architecture (width, straightness, alignment of collagen fibers and collagen molecules inside fibers), increased representation of myofibroblasts and increase expression of collagen-remodelling enzymes (LOX and MMP2). Thus, the changes in organization of collagen fibers, which were already described in the cancer microenvironment, also exist in the mucosa far from the cancer, but smaller in magnitude.

## Introduction

Extracellular matrix (ECM) is no longer considered as an inert substrate, a three-dimensional network which only “fills the spaces” between cells and provide mechanical support^1,2^. Today, ECM is known to be a complex and dynamic structure, whose chemical and biophysical properties affect cell adhesion^3^, proliferation^4^ morphology^5^, migration^6^, regulate tissue morphogenesis^7,8^ and fluid volume in tissues^9^. The most abundant component of ECM in the lamina propria of the colon mucosa is type I collagen.

Remodelling of collagen fibers has been described in almost every solid cancer, including colorectal cancer. During tumor formation and progression, collagen remodelling is constantly carried out: degradation, synthesis, cross-linking of fibers, change of fiber orientation, and interaction of cells of the innate and acquired immune system with collagen fibers^10,11^. Changes in morphology, representation, and organization of collagen fibers contribute to the formation of the microenvironment that favors tumor progression, primarily through its effect on cell migration and polarization^12^. Also, remodelling of collagen fibers on premetastatic sites is of great importance in determination of survival and growth of disseminated cancer cells, and thus, formation of metastasis^13,14^.

Remodelling of collagen fibers may be a result of changes in synthesis, degradation or cross-linking. Main cells responsible for synthesis of collagen in colon mucosa are fibroblasts and myofibroblasts. The most important enzymes for degradation of collagen fibers are matrix metalloproteinases (MMPs). It has been shown that expression of MMP2 and MMP9 is increased in colorectal cancer and influences its progression and metastatic potential^15,16^. Covalent cross-linking of collagen fibrils is catalyzed by enzyme lysyl oxidase (LOX). LOX-dependent collagen crosslinking enhances proliferation of cancer cells and metastatic capacity^17,18^.

The quantification of changes of collagen within the primary tumor and metastatic niches has been the subject of numerous studies and it is recognized to play an important role in both cancer development and progression^19–21^. However, much less is known about remodelling of collagen fibers in healthy looking colon mucosa distant from the cancer. In the previous study of uninvolved colon mucosa^22^, we described changes in the representation and organization of collagen fibers as far as 10 cm and 20 cm away from the colon cancer. Because remodelling of collagen fibers is an important process, crucial for creating specific microenvironmental milieu, we felt that further studies were necessary to investigate the finer aspects of this phenomenon. Thus, the aim of our study was to quantify morphological parameters and organization of collagen fibers and to investigate possible causes of collagen remodelling (change in syntheses, degradation and collagen cross-linking) in the colon mucosa 10 cm and 20 cm away from the cancer in comparison with healthy mucosa. Indeed, we showed that this far from the colon cancer there are changes in collagen architecture, increased representation of myofibroblasts and increase expression of collagen-remodelling enzymes.

## Results

### Changes in morphology and organization of collagen fibers in the uninvolved colon lamina propria visualized using SHG imaging

**Figure 1:**
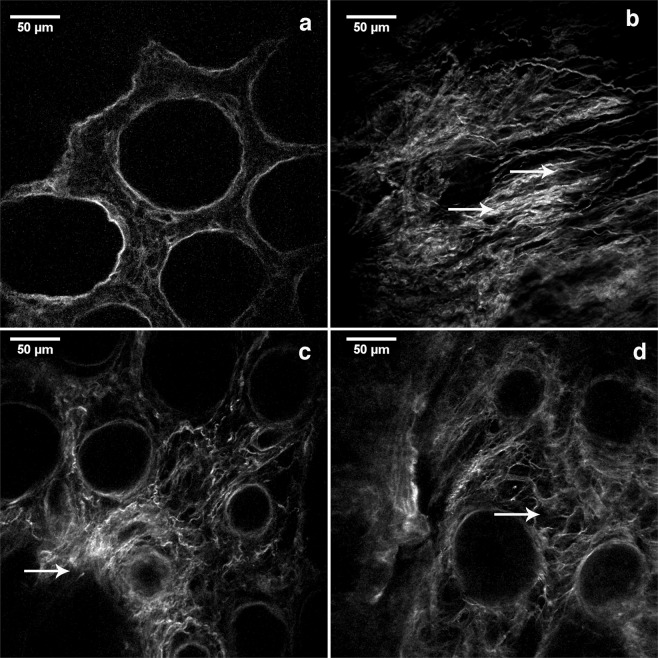
SHG images showing patterns of collagen fibers organization in the lamina propria of colon mucosa in the healthy patients and 10 cm and 20 cm away from the malignant tumor. Collagen fibers were wavy, orderly organized throughout lamina propria and around the crypts in the mucosa of healthy patients (**a**); In the lamina propria at the distance 10 cm (**b**,**c**) and 20 cm away from the cancer (**d**), proper arrangement of collagen fibers was partly lost: regions with parallel collagen fibers (**b**, arrows), thick and dense collagen fibers (**c**, arrow), regions with edema of lamina propria where collagen fibers were separated with large pores (**d**, arrow showing pore) were frequently observed.

On SHG images, in the lamina propria of healthy patients, collagen fibers were wavy, orderly organized throughout lamina propria and around the crypts (Fig. 1a). At the distance 10 cm (Fig. 1b,c) and 20 cm (Fig. 1d) away from the cancer, proper arrangement of collagen fibers appeared partly disturbed. It was possible to observe regions with parallel collagen fibers (Fig. 1b), thick collagen fibers (Fig. 1c), regions with edema of lamina propria where collagen fibers were separated with large pores (Fig. 1d) and regions with fibers organized as in healthy subjects.

**Figure 2:**
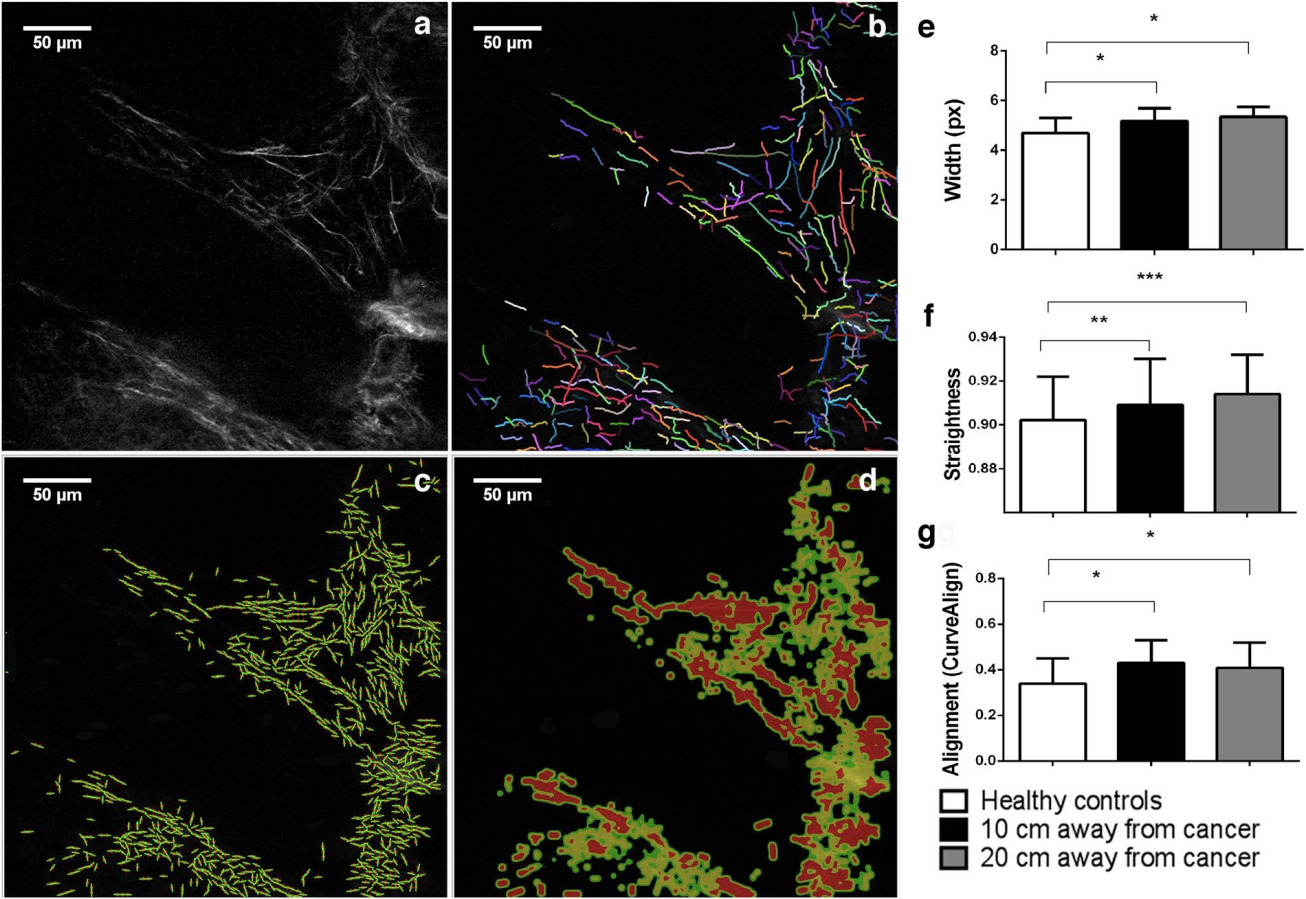
CT FIRE and CURVE Align in analyzing whole SHG images of collagen fiber in the lamina propria of colon mucosa in the healthy patients and 10 cm and 20 cm away from the malignant tumor. Original SHG image of lamina propria of healthy patient. (**a**) Graphical output from CT FIRE showing automatic extraction of collagen fibers, same patient. (**b**) Graphical outputs from CURVE Align for calculating alignment of collagen fibers. (**c**,**d**) Graphs are showing increased width (**e**), straightness (**f**) and alignment (**g**) of collagen fibers in the lamina propria 10 cm and 20 cm away from the cancer, calculated using CT FIRE and CURVE Align. *p < 0.05, **p < 0.001, ***p < 0.0001 (n = 32 healthy patients/96 images and n = 35 cancer patients/105 images; Values are express as mean ± sd; ANOVA).

By analyzing whole SHG images using CT-FIRE software (Fig. 2a,b; 23–25), we have shown that there was a statistically significant increase in the width of collagen fibers in the lamina propria of the colon mucosa at a distance 10 cm (p = 0.032) and 20 cm from the tumor (p = 0.021), compared with healthy subjects (Fig. 2e). Collagen fibers in the lamina propria 10 cm and 20 cm away from the cancer were significantly more straight (p = 0.004 and p < 0.0001, Fig. 2f) compared with collagen fibers in lamina propria of healthy colon. Using CurveAlign software (Fig. 2c,d)^23–25^, based on curvelet transform, it was shown that collagen fibers in colon lamina propria 10 cm and 20 cm away from the cancer were significantly more aligned compared with collagen fibers in healthy lamina propria (p = 0.022 and p = 0.041; Fig. 2g).

**Figure 3:**
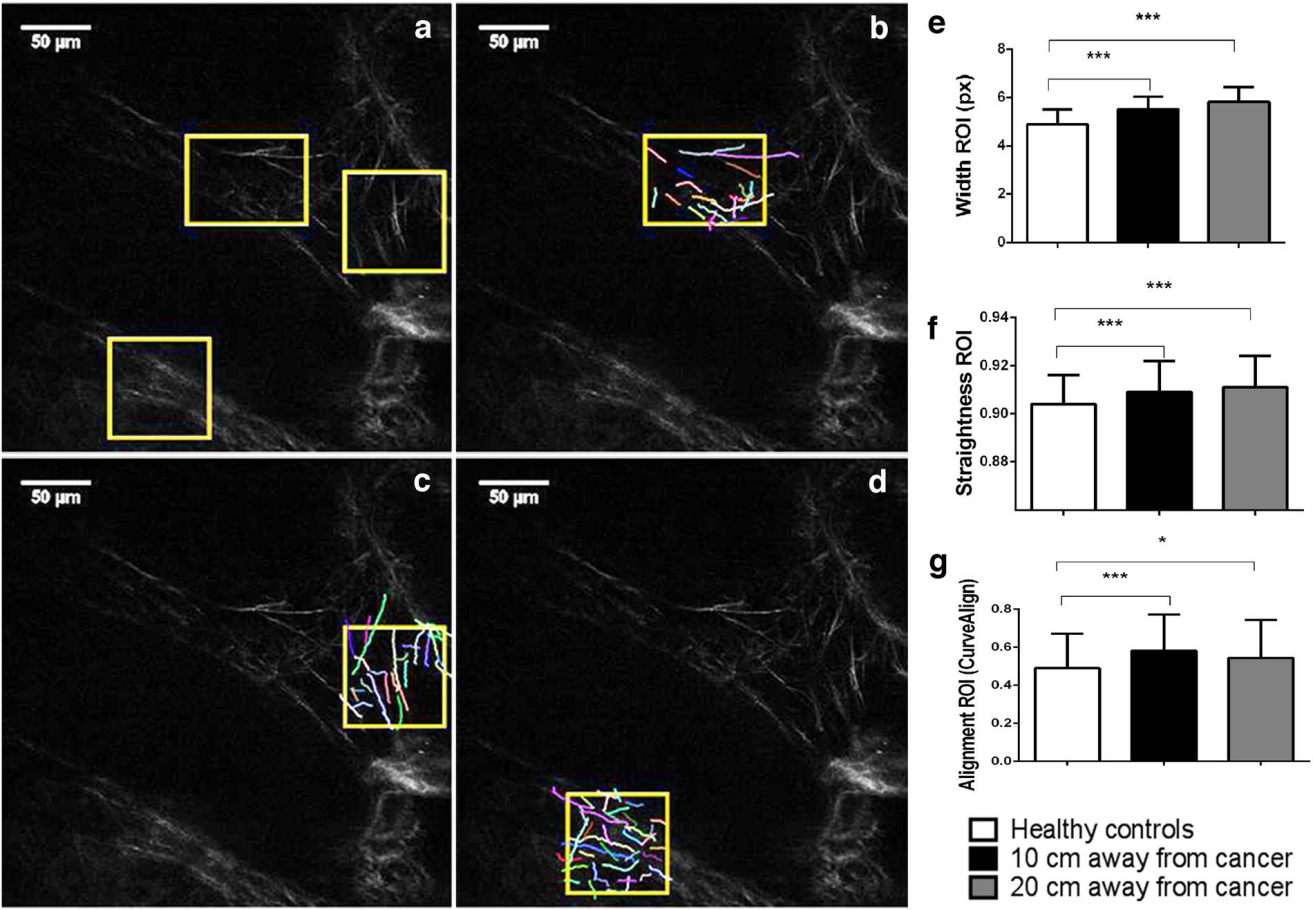
CT FIRE and CURVE Align in analyzing regions of interest (ROIs) on SHG images of collagen fiber in the lamina propria of colon mucosa in the healthy patients and 10 cm and 20 cm away from the malignant tumor. Original SHG image of lamina propria of healthy patient, with labeled rectangular ROIs which include collagen fibers near the Liberkün’s crypts (**a**) and example of CT FIRE collagen fiber extraction within ROIs (**b–d**). Graphs are showing increased width (**e**), straightness (**f**) and alignment (**g**) of collagen fibers in the lamina propria 10 cm and 20 cm away from the cancer, calculated using CT FIRE and CURVE Align. *p < 0.05, **p < 0.001, ***p < 0.0001 (n = 32 healthy patients and n = 35 cancer patients; Values are express as mean ± sd, ANOVA).

Because of the heterogeneity in morphology and organization of collagen fibers in the lamina propria of colon mucosa and according to the studies which showed that the remodelling of collagen fibers within the tumor primarily could be observed in the immediate vicinity of epithelial cells^25,26^, we also performed computational analyses of collagen fibers within 3 regions of interest per each SHG image (Fig. 3a–d). The regions of interest included lamina propria of colon mucosa in the immediate vicinity of Liberkün’s crypts. The observed differences in morphology and organization of collagen fibers, detected by analyzing whole images, were even more pronounced when analysis were conducted inside the regions of interest: At a distance of 10 cm and 20 cm from the tumor, there was a statistically significant increase in width and straightness of collagen fibers compared to lamina propria of colon mucosa of healthy subjects (p < 0.0001, Fig. 3e,f). Also, collagen fibers in colon lamina propria both 10 cm and 20 cm away from the cancer were significantly more aligned compared with collagen fibers in healthy lamina propria (p < 0.0001 and p = 0.035, Fig. 3g).

We also quantified alignment of collagen fibers using another approach. With FiberFit software, based on FFT, we obtained the dispersion parameter *k*^27^. The dispersion parameter *k* was significantly increased 10 cm and 20 cm away from cancer (indicated more aligned collagen fibers), compared with healthy lamina propria (p = 0.031 and p = 0.0013;Table 1).

**Table 1:** Dispersion parameter *k* and anisotropy coefficient β in the lamina propria of colon mucosa in healthy patients and 10 cm and 20 cm away from the cancer.

	Healthy controls (n = 32)	10 cm away from cancer (n = 35)	20 cm away from cancer (n = 35)
*k* dispersion parameter	0.51 ± 0.22	0.91 ± 0.53*	1.11 ± 0.67**
β coefficient	0.26 ± 0.03	0.31 ± 0.04***	0.32 ± 0.05***

^*^p < 0.05, **p < 0.01, ***p < 0.001.

### Changes in SHG polarization anisotropy in the uninvolved colon lamina propria

In the lamina propria of colon mucosa at distance 10 cm and 20 cm away from cancer, anisotropy coefficient β^28,29^ was significantly higher (indicating more orderly organized collagen molecules inside fibrils), compared with lamina propria of healthy patients (p < 0.0001; Table 1).

### Electron microscopy analysis of collagen fibers in the uninvolved colon lamina propria

**Figure 4:**
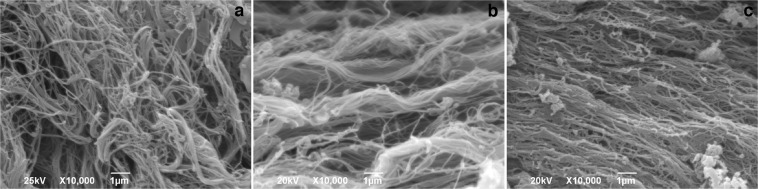
Representative SEM images of collagen fibers in the lamina propria of colon mucosa in the healthy patients (**a**) and 10 cm (**c**) and 20 (**b**) cm away from the malignant tumor. In the lamina propria of healthy patients (**a**) thin collagen fibers were forming relatively dense network. At the distance 10 cm and 20 cm away from the tumor, regions with thick (**b**, 20 cm away from tumor) and aligned collagen fibers (**c**, 10 cm away from tumor) were more frequently observed. Magnification x10 000.

On SEM collagen fibers in healthy patients were thin, curvy, and the network they were forming was relatively dense, with small pores between bundles (Fig. 4a). At the distance 10 cm and 20 cm away from the tumor the thick collagen fibers were more frequently observed (4b). Also, regions with more aligned collagen fibers were alternating with regular, network-like distribution of collagen fibers (Fig. 4c).

### Changes in synthesis, cross-linking and degradation of collagen fibers in the uninvolved colon lamina propria

**Figure 5:**
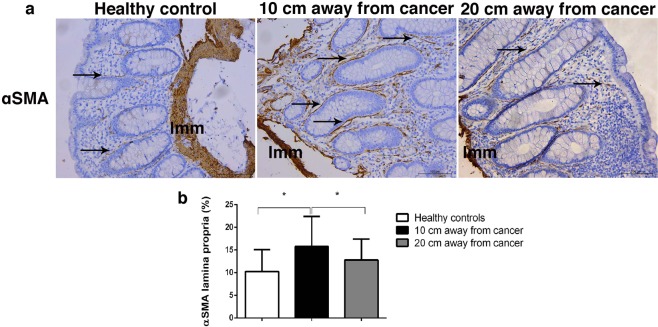
Representative images of αSMA-positive cells in the lamina propria of colon mucosa in healthy patients and at the distance 10 cm and 20 cm away from the tumor. Arrows are showing αSMA-positive myofibroblasts (**a**); Graph is showing increased representation (in %) of αSMA-positive cells in the lamina propria 10 cm and 20 cm away from the cancer, compared with healthy patients. *p < 0.05, **p < 0.001, ***p < 0.0001 (n = 27 healthy patients and n = 30 cancer patients; Values are express as mean ± sd; ANOVA).

Next, we wanted to find out if the changes in morphology and organization of collagen fibers are due to changes in synthesis, cross-linking or degradation of collagen. The main cells involved in collagen synthesis are fibroblast and myofibroblast. We detected myofibroblasts in colon lamina propria, immunohistochemically, using αSMA-antibody. In the lamina propria of healthy patients, myofibroblasts formed continuous layer around crypts, with few αSMA-positive cells throughout lamina propria (elongated, spindle-shaped, most probably also myofibroblasts) and around blood vessels (smooth muscle cells) (Fig. 5a). At the distance 10 cm and 20 cm away from the cancer, pericryptal myofibroblast were readily identifiable, forming thicker-appearing layer. More αSMA positive cells were visible throughout lamina propria (Fig. 5a). Quantitative analysis, using Color Picker Threshold plugin, showed significantly higher representation of αSMA-positive cells 10 cm away from cancer, compared with healthy lamina propria and lamina propria at the distance 20 cm away from the cancer (p = 0.018 and p = 0.037) (Fig. 5b).

**Figure 6:**
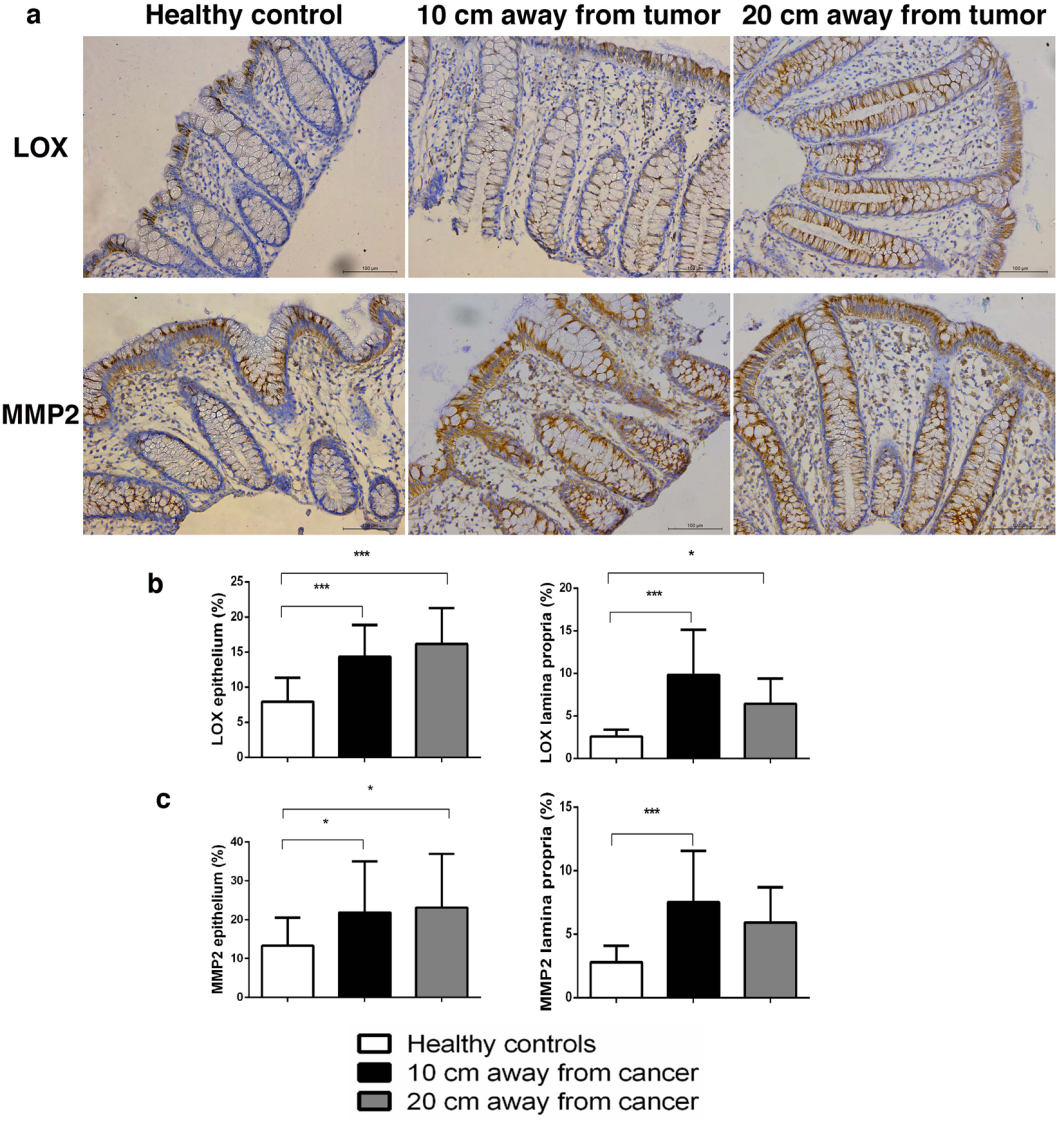
Representative images of LOX and MMP2 staining in the epithelium and lamina propria of colon mucosa in healthy patients and at the distance 10 cm and 20 cm away from the tumor (**a**); Graphs are showing increased representation (in%) of LOX (**b**) and MMP2-positive cells (**c**) in the lamina propria 10 cm and 20 cm away from the cancer, compared with healthy patients; *p < 0.05, **p < 0.001, ***p < 0.0001 (n = 27/28 healthy patients and n = 30 cancer patients; Values are express as mean ± sd; ANOVA).

Lysyl Oxidase (LOX) catalyzes crosslinking of collagen molecules during collagen fibrils assembly. We detected LOX expression in epithelial cells (both surface epithelium and epithelium of Lieberkühn glands) and in lamina propria of colon mucosa (Fig. 6a). In the colon epithelial cells, LOX mainly showed perinuclear expression pattern. LOX expression was significantly higher in epithelial cells of colon mucosa 10 cm and 20 cm away from the cancer, compared with healthy mucosa (p < 0.0001; Fig. 6b). In colon lamina propria, LOX was predominantly expressed by fibroblasts and myofibroblasts and subepithelial macrophages (Fig. 6a). LOX expression was significantly higher in lamina propria of colon mucosa 10 cm and 20 cm away from the cancer, compared with healthy controls (p < 0.0001 and p = 0.013; Fig. 6b).

Matrix metalloproteinases play an important role in degradation of ECM including collagen fibers. Their expression is changed in colon cancer^15^. We wanted to find out if MMPs were involved in remodelling of collagen fibers this far from the colon cancer. MMP2 in the colon epithelial cells showed supranuclear expression. Some intraepithelial lymphocytes were also MMP2-positive (Fig. 6a). MMP2 expression was significantly higher in epithelial cells of colon mucosa 10 cm and 20 cm away from the cancer, compared with healthy mucosa (p = 0.037 and p = 0.034; Fig. 6c). MMP2 was expressed by mononuclear cells in colon lamina propria (Fig. 6a). MMP2 expression was significantly higher in lamina propria of colon mucosa 10 cm away from the cancer, compared with healthy controls (p < 0.0001; Fig. 6c).

MMP9 was barely detectable both in healthy colon mucosa and 10 cm and 20 cm away from the cancer: only few scattered cells through lamina propria, most likely macrophages, were MMP9 positive (Supplementary Fig S1).

## Discussion

Our work demonstrated the changes in morphology and organization of collagen fibers in the colon mucosa 10 cm and 20 cm away from the cancer and provided a brief insight into the possible causes of the collagen remodelling.

Intriguingly, these changes were already described in the cancer microenvironment but larger in magnitude^15,23,25,30,31^.

Change in deposition, alignment and cross-linking of collagen fibers, influence cell polarity and cell-cell interactions, increases growth factor signaling and stimulate migration of cancer cells^2,32^. Cells are able to sense and response to changes of both biochemical and biomechanical properties of the local microenvironment. Some of the main parameters determining biomechanical properties of collagen network are thickness of the fibers, alignment, stiffness and porosity. Increasement of collagen fibers thickness was shown to correlate with formation of invadopodia, change in cancer cells shape and increase migratory capacity^2,33^. Increased alignment of collagen fibers has significant impact on gene expression, differentiation, proliferation and especially migration of cancer cell, with align collagen fibers acting as “highways” for cancer cell migration^2^. Stiffness is strongly related to LOX-induced cross-linking of collagen fibers, which as a consequence favors cell adhesion and MMPs secretion^2,34,35^.

More recently, the importance of tissue away from the cancer is being recognized, and the number of papers investigating changes in the uninvolved tissue, on genetic, epigenetic, biochemical and structural level, is increasing^36–38^. The most studied was the uninvolved mucosa immediately around the colon cancer, commonly up to 2 cm away from the cancer, so called transitional mucosa^39^. Recent studies showed that there are localized densification and increased alignment of collagen fibers in the transitional mucosa immediately around the cancer^40^. Rare groups of authors also analyzed healthy looking mucosa further from the cancer: Roy and colleagues have described changes in the rectal mucosa of patients bearing advanced adenomas elsewhere in the colon: metabolic reprograming, including evidence of Warburg effect, early increase in microvascular blood supply and also, increased cross-linking and local alignment of collagen fibers^36,38^. They have also shown that increased cross-linking of collagen in the uninvolved colon mucosa fibers was due to increased expression of LOX enzyme^36^. Using microarray, qRT-PCR and immunohistochemistry Trujillo *et al*. have demonstrated changed gene expression signature in the tissue 1 cm and 5 cm away from the breast cancer: differentially expressed genes were involved in extracellular matrix remodelling, including genes for MMPs, wound healing, fibrosis and epithelial to mesenchymal transition^41^. Sanz-Pamplona et. al. revealed number of genes that were preferentially activated in adjacent mucosa from colorectal cancer, compared with mucosa of healthy patients: among other, these were genes involved in TGF-beta signaling pathway which is associated with fibrosis, genes for MMPs, cell adhesion molecules, cell-ECM integrin signaling pathways and BMP2 signaling pathways^37^.

So, our results are consistent with and complement the works cited: Genes involved in ECM remodelling are differentially express in the mucosa around the cancer^41^, and, by analyzing morphology, organization and cellular composition in the colon mucosa far from the cancer, we showed the consequences of these altered gene expression.

We have analyzed uninvolved colon mucosa quite far from the colon cancer, 10 cm and 20 cm away, respectively. Most authors still consider tissue located more than 5 cm away from the colon cancer completely healthy and use it as a control in their research^42^. We had at least two reasons to believe that, although distant, this tissue could also bear changes: the systemic effects of tumors and, so-called, field carcinogenesis effect^36,43,44^.

More recently, very interesting concept has emerged, according to which tumor initiation and progression are shaped by body’s systemic response to tumor, which implied involvement of distant, uninvolved tissues and organs. Tumor produce a vast number of cytokines (for example, VEGF-A, TGF-β, TNF-α) and extrude different microvesicles, which act in a systemic fashion, modulating the behavior of host cells in distant tissues, most notably bone marrow, spleen and pre-metastatic niches. So, by secreting cytokines, tumor induce changes in distant tissues, which lead to the formation of local microenvironment that makes that particular tissue more permissive for seeding and survival of metastatic cancer cells^43^. Remodelling of extracellular matrix play particularly important role in creating microenvironment permissive for metastatic cancer cells: activation of fibroblasts/myofibroblasts, reorganization of collagen fibers, and change in expression of ECM-remodelling enzymes such as MMP2, MMP9 and LOX^43,45^.

On the other hand, according to the field carcinogenesis concept, environmental carcinogens and genetic risk factors act on the entire organ (in our case, entire colon mucosa) leading to the emergence of an altered field, so called “field of injury”. On this altered field, additional stochastic genetic and epigenetic events within the enabling microenvironment could give rise to focal cancers^36,44^. ECM, especially collagen and myofibroblasts, is recognized to play an important role in the field carcinogenesis concept by participating in the formation of enabling microenvironment. It is believed that altered epithelial cells induce change in the surrounding microenvironment that, in turn, promote or modify expansion of altered cells, or, there are even evidence indicating that ECM changes could play a primary role in both cancer initiation and progression^46^.

Whether the changes in morphology and organization of collagen fibers 10 cm and 20 cm away from the cancer, represent a consequence of a growing tumor or a field effect, or a combination, we have no answer. Further analyses of mucosa more distant from the cancer are needed. Subsequently, detailed analyses of changes in the epithelium (genetic, epigenetic, biochemical and morphological) this far from the cancer and analysis of epithelial-stromal interactions on molecular level would be the next step in more thorough understanding of complex ways in which cancer interact with surroundings, distant parts of the same organ and systemically, with distant tissues and organs. Also, it would be important to conduct described analyses on the larger number of patient- to explore their potential in colon cancer screening or stratification of patients for colonoscopy.

## Materials and methods

### Tissue samples

Tissue samples were obtained during colonoscopy at the Department of gastrointestinal endoscopy, University Hospital Center “Dr Dragiša Mišovic-Dedinje”, Belgrade, Serbia, from patients suspected to suffer from colon cancer based on clinical symptoms. When the experienced gastroenterologist noticed a suspected change during colonoscopy, they took samples of unaffected colon mucosa 10 cm and 20 cm away in caudal direction. The samples of unaffected colon mucosa were obtained from 41 patients older than 50 years (24 males and 17 females; Table 2). Only tissue samples for which pathologist confirmed that the suspected change was colorectal adenocarcinoma, were included in the study. For all patients, it was newly discovered cancer, so they haven’t been on any kind of treatment for the malignant disease before.

**Table 2:** Demographic characteristics of patients included in the study.

Patients	Number	Age (years)	Gender	
			Male	Female
Cancer	41	74.3	24	17
Healthy	39	71.9	20	19

As a control, the samples of colon mucosa were collected in the same institution, from 39 patients (20 males and 19 females; Table 2) who were indicated colonoscopy because of rectal bleeding, anemia or weight loss, and were without any pathological finding or diagnosed only with uncomplicated hemorrhoids (Haemorrhoides non specificatae sine complicationibus). Patients with inflammatory bowel disease, infectious colitis or diverticular disease of colon were excluded from the study. Our study was approved by the Ethics Committee of University Hospital Center “Dr Dragiša-Mišović-Dedinje”, Belgrade, Serbia (18/10/2017). All methods were carried out in the accordance with relevant guidelines and regulations.

### Second harmonic generation imaging of colon tissue samples

The images of collagen fibers in the label-free human colon tissue samples were obtained using an original lab frame nonlinear laser-scanning microscope^47,48^. For second harmonic generation (SHG) imaging of collagen fibers following experimental setup for nonlinear laser scanning microscope (NLM) was used^22^: The tunable mode-locked Ti:sapphire laser (Coherent, Mira 900) has been source of the infrared femtosecond pulses. The laser light was directed onto the sample by a short-pass dichroic mirror (cut-off at 700 nm) through the Zeiss EC Plan-Neofluar 40×/1.3 NA Oil objective. The laser wavelength was 840 nm. The SHG was detected in back-reflection arm. The narrow bandpass filter at 420 nm (Thorlabs FB420-10, FWHM 10 nm) blocks the scattered laser light and auto-fluorescence, and passes second harmonic at 420 nm. The average laser power on the sample was 30 mW. According to the pulse duration (160 fs) and repetition rate (76 MHz), we estimate the peak laser power to be 2.5 kW.

### Quantitative analysis of collagen fibers in colon lamina propria

To analyze morphology and organization of collagen fibers in colon lamina propria, on SHG images, we used two complementary morphology based and one morphology-independent approach. For morphological assessment of collagen fibers we used methods based on curvelet transform and Fourier transform. As a morphology-independent approach, we measured SHG polarization anisotropy.

#### Computational collagen fiber quantification

CT-FIRE, an open-source software package, was used for calculation of width and straightness of collagen fibers. CT-FIRE was developed to automatically extract and analyze individual collagen fibers from SHG images^23–25^. Widths of collagen fibers are expressed in pixels. Straightness is represented on a scale 0–1, where 1 corresponds to perfectly straight fibers. CURVE Align software was used to calculate alignment of collagen fibers. Alignment was represented on a scale from 0–1, where 1 indicates all fibers orientated at the same angle^23–25^. CT-FIRE and CURVE Align measurements were applied both on whole images (from 32 healthy patients and 35 cancer patients, 3 SHG images per patient) and on 3 regions of interest (300 × 300 px^2^) per image, located in the close vicinity to Liberkün glands.

An additional software, FiberFit, which is based on fast Fourier transforms (FFT) was use to quantify orientation of collagen fibers in colon tissue samples (from 32 healthy patients and 35 cancer patients, 3 SHG images per patient). Using FiberFit, we obtained the dispersion parameter *k*, used to quantify collagen fiber alignment (low *k* values indicates disordered networks, large *k* values indicates aligned networks^27^.

#### SHG polarization anisotropy

The SHG anisotropy could be used to quantify alignment of collagen molecules inside fibers. The anisotropy parameter β was calculated by:

$$\beta =({I}_{{\rm{par}}}-{I}_{{\rm{orth}}})/({I}_{{\rm{par}}}+2\cdot {I}_{{\rm{orth}}})$$

where *I*_par_ and *I*_orth_ represented SHG intensity detected when the analyzing polarizer is oriented parallel (I_*par*_) and orthogonal (I_*ort*_) to the laser polarization^28,29^. Values of β range from 0 to 1, where 0 represents completely random and 1 completely aligned collagen molecules inside fibers.

We analyzed 32 samples from healthy patients and 35 from cancer patients. From each tissue sample 3 randomly chosen regions on magnification x400 were measured.

### SEM analysis of collagen fibers in colon lamina propria

The surface morphology of collagen fibers (for 7 healthy patients and 6 cancer patients) has been examined using a JEOL JSM-6390LV SEM (JEOL, Japan) at an accelerating voltage of 10 kV. After fixation in 3% glutaraldehyde in cacodulte buffer, dehydration in graded alcohols (50%, 70%, 96%, 100%, 100%) the colon tissue samples were immediately dried using Critical Point Dryer K850 (Quorum Technologies, Laughton, UK). Prior to visualization, the dry samples were sputtered with gold using a Bal-Tec SCD 005 Cool putter Coater.

#### Immunohistochemistry

Immunohistochemical analysis was performed on formalin-fixed, paraffin-embedded sections using following antibodies and dilution ratios: anti-alphaSMA (Dako, M0851 1:500), anti-MMP9 (Abcam, ab38898, 1:500), anti-MMP2 (Abcam, ab37150, 1:500), anti-LOX (Abcam, ab174316, 1:500) (Table 3). After heat-induced antigen retrieval using citrate buffer (pH = 6) and subsequent washing in PBS, primary antibodies were incubated for 60 minutes. The sections were treated with commercial Ultra Vision/3,3’-diaminobenzidine (DAB) staining kit (Thermo Scientific Lab Vision TL-060-HD, Rockford, IL, USA). The reactions were developed using DAB substrate.

**Table 3:** The number of patients analyzed and the list of antibodies used for immunohistochemical analysis of myofibroblast, MMPs and LOX in the healthy lamina propria, 10 cm and 20 cm away from the cancer.

	Number of analyzed healthy patients	Number of analyzed cancer patients (tissue away from the cancer)	
		10 cm away	20 cm away
αSMA (Dako, M0851)	27	30	30
LOX (Abcam, ab174316)	27	30	30
MMP2 (Abcam, ab37150)	28	30	30
MMP9 (Abcam, ab38898)	12	15	13

For quantification of immunohistchemically stained sections, Color Picker Threshold plugin within open community platform for bioimage informatics Icy was used, as previously described^22^. On images stained with anti-alphaSMA antibody, the representation of myofibroblast in colon lamina propria was determined as a relative percentage of the area occupied by myofibroblast divided by the area of the lamina propria selected with an imaging processor. For slides stained with anti-MMP2 and anti-LOX antibody, the percentage of MMP2/LOX-positive area was determined separately in lamina propria and epithelial region. The number of analyzed patients for each antibody is in the Table 3. For one patient, one slide was stained with each antibody and a random selection of 10 fields per slide on magnification x200 was analyzed.

### Statistical analysis

Data were presented as means and standard deviations. The statistical package SPSS for Windows 12.0 (SPSS inc., Chicago, IL, USA) was used to indicate significant differences (two-way ANOVA followed by Tukey’s multiple comparison test). Statistical significance was determined by p < 0.05.

## Supplementary information

Supplementary information.

## Data Availability

The dataset generated during and/or analyzed during the current study are available from the corresponding author on reasonable request.
