# Supplementary material for: Altered organization of collagen fibers in the uninvolved human colon mucosa 10 cm and 20 cm away from the malignant tumor
Source: Sci Rep. 2020 Apr 14;10:6359. doi: 10.1038/s41598-020-63368-y (PMC7156654; doi:10.1038/s41598-020-63368-y)
Supplement: Supplementary file 1 — Supplementary information. [file 41598_2020_63368_MOESM1_ESM.docx]

Altered organization of collagen fibers in the uninvolved human colon mucosa 10 cm and 20 cm away from the malignant tumor

Sanja Z. Despotović^1*^, Đorđe N. Milićević^2^, Aleksandar J. Krmpot^3^, Aleksandra M. Pavlović^4^, Vladimir D. Živanović^4^, Zoran Krivokapić^5^, Vladimir B. Pavlović^6^, Steva Lević^6^, Gorana Nikolić^7^, Mihailo D. Rabasović^3^


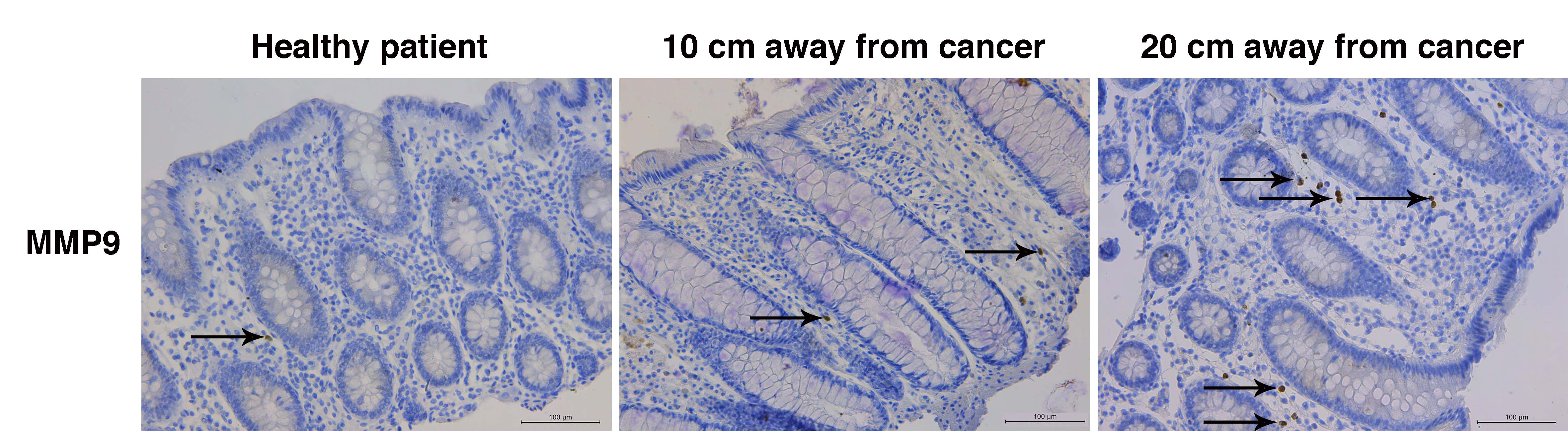


Supplementary Figure S1. Expression of MMP9 in the colon mucosa of the healthy patients and 10 cm and 20 cm away from the malignant tumor. Arrows are showing some of the rare MMP9-positive cells in colon lamina propria. (12 healthy patients, 15 samples 10 cm away from tumor and 13 samples 20 cm away from tumor).
